# Roles of NAD(P)H:quinone Oxidoreductase 1 in Diverse Diseases

**DOI:** 10.3390/life11121301

**Published:** 2021-11-26

**Authors:** Wang-Soo Lee, Woojin Ham, Jaetaek Kim

**Affiliations:** 1 Division of Cardiology, Department of Internal Medicine, College of Medicine, Chung-Ang University, Seoul 06974, Korea; 2 Division of Endocrinology and Metabolism, Department of Internal Medicine, College of Medicine, Chung-Ang University, Seoul 06974, Korea; dglyue5@cau.ac.kr

**Keywords:** NAD(P)H:quinone oxidoreductase 1, oxidative stress, human diseases

## Abstract

NAD(P)H:quinone oxidoreductase (NQO) is an antioxidant flavoprotein that catalyzes the reduction of highly reactive quinone metabolites by employing NAD(P)H as an electron donor. There are two NQO enzymes—NQO1 and NQO2—in mammalian systems. In particular, NQO1 exerts many biological activities, including antioxidant activities, anti-inflammatory effects, and interactions with tumor suppressors. Moreover, several recent studies have revealed the promising roles of NQO1 in protecting against cardiovascular damage and related diseases, such as dyslipidemia, atherosclerosis, insulin resistance, and metabolic syndrome. In this review, we discuss recent developments in the molecular regulation and biochemical properties of NQO1, and describe the potential beneficial roles of NQO1 in diseases associated with oxidative stress.

## 1. Introduction 

In mammals, the NAD(P)H:quinone oxidoreductase (NQO) family comprises two flavin adenine dinucleotide (FAD)-dependent flavoproteins: NQO1 and NQO2 [1]. NQO1 was first identified in the rodent liver, and was designated as DT-diaphorase [2], whereas NQO2 was first isolated and characterized by screening a human liver cDNA library via hybridization with an NQO1 cDNA probe [3,4]. NQO1 and NQO2 are highly expressed in mammalian organs, with the greatest abundances detected in the liver, kidneys, and cardiovascular system [1,3,4]. In humans, *NQO1* and *NQO2* are located on chromosomes 16q22.1 and 6pter-q12, respectively [1,5]. NQO1 and NQO2 are ubiquitous cytosolic enzymes with molecular weights of 31 and 25 kDa, respectively [1], and their crystal structures enable functional and structural comparisons between the two enzymes [3,6,7,8,9,10,11]. The potential roles of NQO1 in chemoprotection have been widely reviewed [6,12,13,14,15,16], and the mechanisms and structure of NQO1 have been extensively discussed [6,11,17,18]. Importantly, both NQO1 and NQO2 catalyze the two-electron reduction of diverse endogenous and exogenous quinone compounds and their derivatives, leading to the prevention of redox cycling because of one-electron reduction as well as detoxification of electrophilic compounds [1,5].

Oxidative stress (OS) refers to an imbalance status between oxidants and antioxidants, in which the influence of oxidants is dominant [19]. Intracellular reactive oxygen species (ROS)—such as superoxide (·O_2_^−^) and hydrogen peroxide (H_2_O_2_)—cause OS when their abundance become excessive [19]. Evidence from clinical and experimental studies has shown that inflammation and OS are associated with diverse disease entities, such as atherosclerosis, cardiovascular disease, obesity, hypertension, diabetes mellitus, ischemia–reperfusion injury, cancer, and Alzheimer’s disease (AD) [20,21,22,23,24,25]. Moreover, several recent studies have demonstrated the promising roles of NQO1 in protecting against cardiac and vascular damage, as well as related diseases such as dyslipidemia, atherosclerosis, insulin resistance, and metabolic syndrome [1,6,19]. 

In this review, we summarize recent developments in the molecular regulation and biochemical properties of NQO1, and describe the potential beneficial roles of NQO1 in OS-related diseases.

## 2. Substrates and Induction of NQO1

**Figure 1:**
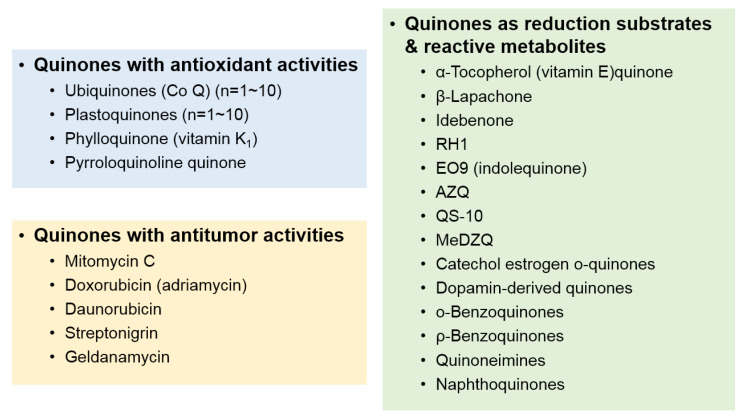
Schematic diagram of NQO1 substrates. Abbreviations—Co Q: coenzyme Q; RH1: 2,5-diaziridinyl-3-hydroxymethyl-6-methyl-1,4-benzoquinone; AZQ: diaziquine; QS-10: 6-(9-carboxynonyl)-2,3-dimethoxy-5-methyl-1,4-benzoquinone; MeDZQ: 2,5-diaziridinyl-3,6-dimethyl-1,4-benzoquinone.

Many exogenous compounds and xenobiotic quinones are metabolized by NQO1 [12,14,26,27], whereas only a few endogenous quinones are metabolized by NQO1. Some of the representative substrates metabolized by NQO1 are shown in Figure 1 [28,29,30,31,32,33,34].

Induction of NQO1 is developed as a component of the nuclear factor erythroid 2-related factor 2 (Nrf2)-induced adaptive response to cellular stresses, such as oxidation and electrophilic stress, and is activated as part of the aromatic hydrocarbon receptor (AhR)-induced response [16,35]. Moreover, the methylation status of the NQO1 promoter may be an essential element in regulating NQO1 expression [26,36,37,38,39]. Other mechanisms for the transcriptional modulation of NQO1 by anti-estrogen-liganded estrogen receptors have been also proposed [16,40,41]. To elaborate on the induction of NQO1, its main mechanism is associated with the Kelch-like ECH-associated protein 1 (Keap1)/Nrf2/antioxidant response element (ARE) system regulating the expression of several phase-II detoxifying enzyme genes [1,6,26,42]. The Keap1/Nrf2 system helps in the protection of mammalian cells from many xenobiotics. Activation of Nrf2/ARE signaling is differentially modulated during chronic and acute stress [26]. Under normal conditions, Keap1 maintains Nrf2 in the cytoplasm, and stimulates its degradation via ubiquitination. During acute OS, oxidized molecules adjust the interaction of Keap1 and Nrf2 by mediating translocation of Nrf2 from the cytoplasm to the nucleus, where it can bind to the ARE. This stimulates the expression of detoxification and antioxidant genes [26,42]. Thus, the Keap1/Nrf2/ARE system pathway is regarded as the master regulator of cytoprotective responses against OSs and electrophilic xenobiotics [42].

## 3. Biological Role of NQO1

**Figure 2:**
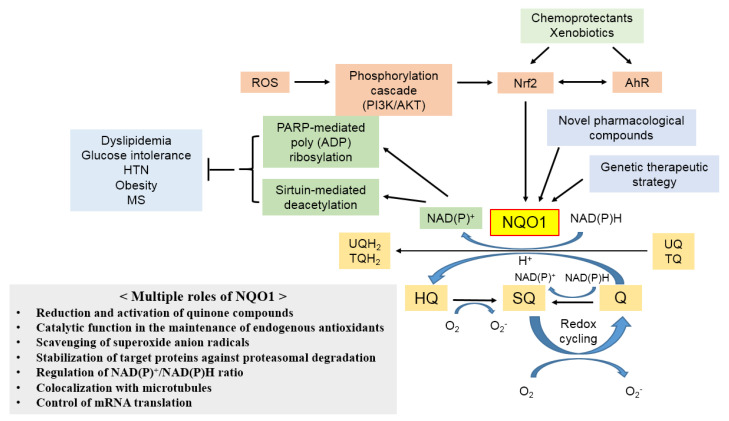
Schematic illustration of the induction and the multiple roles of NQO1. Chemoprotectants and xenobiotics may lead to expression of the *NQO1* gene via the activation of AhR, Nrf2, or both. NQO1 catalyzes the two-electron reduction of quinones; it escapes the one-electron reduction and avoids subsequent redox cycling of the quinone derivatives and the formation of reactive oxygen species. Both gene therapy and novel pharmacological compounds may be applied to elevate the expression of NQO1 in cardiac and vascular systems. Upregulation of NQO1 may be a potential therapeutic strategy for cardiovascular protection from oxidative stress and inflammation. Oxidation of NADH/NADPH through the activation of NQO1 has been found to protect against dyslipidemia, glucose intolerance, hypertension, obesity, and metabolic syndrome. Other signaling pathways may also be associated with the effects of NQO1 on cardiovascular diseases. Abbreviations—ROS: reactive oxygen species; PI3K: phosphoinositide 3-kinase; AKT: serine/threonine-specific protein kinase; Nrf2: nuclear factor erythroid 2-related factor 2; AhR: aromatic hydrocarbon receptor; HTN: hypertension; MS: metabolic syndrome; PARP: poly (ADP-ribose) polymerase; NQO1: NAD(P)H:quinone oxidoreductase 1; Q: quinone; SQ: semiquinone; HO: hydroquinone; UQ: ubiquinone; TQ: α-tocopherol quinone; UQH_2_: hydroubiquinone; TQH_2_: α-tocopherol hydroquinone.

NQO1 has several functions and biological roles, including roles in the reduction and activation of quinone compounds and their derivatives, maintenance of endogenous antioxidants, stabilization of proteins against proteasomal degradation, generation of NAD^+^, colocalization with microtubules, and control of mRNA translation, as illustrated in Figure 2. These multiple roles of NQO1 affect the development and progression of a diverse range of diseases. Briefly described, oxidation of NADH/NADPH through the activation of NQO1 has been found to protect against dyslipidemia, glucose intolerance, hypertension, obesity, and metabolic syndrome. Other signaling pathways, such as Nrf2, may also be associated with the effects of NQO1 on cardiovascular diseases. The detailed pathophysiological mechanisms of NQO1 for various diseases are described in Section 6.

### 3.1. Reduction and Activation of Quinone Compounds and Their Derivatives

The most extensively studied role of NQO1 is its protective action against the harmful arylating and oxidative activities of quinones; numerous studies on this topic have utilized menadione in cellular and animal model systems [26,43,44,45,46,47,48]. NQO1 catalyzes the two-electron reduction and detoxification of quinones and their compounds. By catalyzing two-electron reductions, NQO1 converts quinones directly into hydroquinones and bypasses the one-electron-reducing pathways, catalyzed by enzymes such as cytochrome P450 reductase, which produce semiquinone intermediates that lead to ROS generation via redox cycling [26,49,50]. These ROS can contribute to cytotoxicity and bioactivation. The NQO1 enzyme also catalyzes the two-electron reduction of nitroaromatics, azo dyes, and quinoneimines [1,5].

Contrary to this action of NQO1, the generation of unstable forms of hydroquinone in the metabolism of naturally derived or synthesized quinone substances has also been observed [11,26,36,37]. Moreover, superoxide dismutase can also affect the autoxidation rates of hydroquinones produced by NQO1, and can either accelerate or inhibit autoxidation, depending on the redox chemistry and stability of the hydroquinone [15,26]. These quinone compounds and derivatives are referred to as bioreductive substances, and the activation of quinones and unstable hydroquinones generated during their metabolic processes has been used as a common approach in antitumor drug design [51,52,53,54]. NQO1-catalyzed production of reactive hydroquinones derived from numerous compounds—such as deoxynyboquinone, β-lapachone, mitomycin C, E09, streptonigrin, geldanamycin-based heat-shock protein 90 inhibitors, and aziridinyl benzoquinones—has been utilized to kill NQO1-rich solid tumor cells [54,55,56,57,58,59,60,61,62,63,64,65]. Through this, it was confirmed that the function of NQO1 is not limited to detoxification, but has a two-sided nature that causes detoxification or toxicity for each different substrate, which was presented as a target for cancer treatment. Therefore, the efficient NQO1-mediated reduction of quinones to chemically reactive hydroquinones has been manipulated in numerous innovative studies designed to target drugs to various tumors expressing high of levels NQO1. Several quinone conjugates have been generated which, following hydroquinone formation, undergo chemical rearrangement and trigger the release of cytotoxic molecules from the quinone components [66,67,68,69,70,71,72,73].

### 3.2. Catalytic Function in the Maintenance of Endogenous Antioxidants

A previous study has proposed that NQO1 may play a specific antioxidant role, mainly via the reduction of endogenous quinones, which protects cellular membranes from lipid peroxidation [74]. NQO1 defends the cell against unwanted oxidation by maintaining the reduced form of endogenous antioxidants. Primarily, both ubiquinone (co-enzyme Q (CoQ)) and α-tocopherol quinone (TQ)—two essential lipid-soluble antioxidants—are substrates for NQO1 in vitro. NQO1 catalyzes the two-electron reduction of CoQ and TQ to their hydroquinone forms: hydroubiquinone (UQH or CQH) and α-tocopherol hydroquinone (TQH), respectively [1,74]. Previous research has revealed that NQO1 can stimulate the reduction of CoQ analogs to their reduced ubiquinol forms in liposomes and hepatocytes in a rat model [6,28]. Generally, the decreased rate of CoQ derivatives is dependent on chain length, with molecules containing longer chains—such as CoQ9 or CoQ10—being less efficiently reduced. In the aforementioned research, it was established that the reduced forms of CoQ9 or CoQ10 formed following reduction by NQO1 are effective antioxidants that can defend membrane phospholipids from lipid peroxidation. Moreover, the same research demonstrated that CoQ10 protects against loss of membrane permeability, and that protection against membrane damage after treatment with Adriamycin could be blocked by NQO1 inhibitors. Together, along with its role in the electron transport chain, CoQ also works as an antioxidant enzyme, and is found in most cell membranes in its reduced form. NQO1 was selected as a CoQ reductase during evolution, and NQO1-mediated conversion of other synthetic molecules and xenobiotics may be secondary to its primary effects on CoQ [6,28]. NQO1 is involved in the metabolism of α-tocopherol (vitamin E) derivatives. TQ, a product of α-tocopherol oxidation, has been revealed to have antioxidant capacities following reduction to TQH [75,76,77]. In previous studies, it was demonstrated that NQO1 is active against TQ, promoting its reduction to TQH, and this effect protects against damage to the cell membrane [6,76,77]. Another study also showed that Chinese hamster ovary (CHO) cells transfected with various levels of human NQO1 produced higher levels of TQH, and CHO cells producing higher levels of THQ were more resistant to lipid peroxidation than NQO1-knockout cells [29]. The protective role of NQO1 from oxidation has also been confirmed in human studies, wherein the NQO1 protein was expressed in diverse tissues, such as epithelial cells of the lungs, breast, and colon, offering a significant level of protection against oxidative stresses. The high levels of NQO1 demonstrate that NQO1 may play an essential antioxidant role in these cells [6,29,65].

### 3.3. Scavenging of Superoxide Radicals

NQO1 may also contribute directly to antioxidant protection [1,6,78]. Indeed, NQO1 is known to inhibit the formation of ROS via the redox cycling of diverse quinone derivatives and their compounds. Moreover, NQO1 was recently shown to directly eliminate superoxide in a pyridine-nucleotide-dependent manner [1,78]. This effect may be particularly important in tissues in which NQO1 is highly expressed, such as blood vessels and the myocardium [64,74,79,80]. Evidence for the induction of NQO1 by OS is observed following exposure to ultraviolet radiation and X-rays, which generate OS and can induce as much as a 30-fold increase in NQO1 expression in human cells [6,81]. Studies have confirmed the catalytic activity of NQO1 as a superoxide reductase directly, using electron paramagnetic resonance spectroscopy and a variety of superoxide-generating systems [78,79].

### 3.4. Stabilization of Target Proteins 

A potentially relevant property of NQO1 is its capacity to interact with other proteins, and several of such interactions have been shown to be involved in cellular function [6]. NQO1 has been shown to play important roles in stabilizing the p53 protein via steps such as protein–protein interactions, which inhibit ubiquitin-independent degradation of p53 by the 20S proteasome [1,82,83]. This mechanism may explain the lower basal levels of the p53 protein studied in the animal model of NQO1-null mice [84]. NQO1 has been shown to stabilize an increasing number of proteins, including ornithine decarboxylase, p53, p63, p73, and peroxisome proliferator-activated receptor-γ coactivator-1α (PGC1α), in an NAD(P)H-dependent manner [6,85,86,87]. The proteins that interact with NQO1 are diverse, and a recent study revealed the NQO1-induced stabilization of hypoxia-inducible factor-1α (HIF-1α) in colon tumors from murine xenografts, via the mechanism of impaired proteasomal HIF-1α degradation [88]. This finding suggests that targeting the stability of NQO1—leading to destabilization of HIF-1α—may be a good option as a novel anticancer strategy. Stabilization of p53 by NQO1 may exert protective effects against carcinogenesis [1,6]. Moreover, because p53 is also associated with modulation of apoptosis in vascular cells, stabilization of the p53 protein by extremely expressed NQO1 in the heart and blood vessels may contribute to protection against cardiovascular pathophysiology-related dysregulation of apoptosis [1,26]. In addition to p53, NQO1 has also been shown to regulate the ubiquitin-independent 20S proteasomal degradation of p33 and p73α—two other tumor suppressors [1,89,90]. In murine liver tissue, NQO1 interacts with the 20S proteasome, and NQO1 may act as a gatekeeper of the 20S proteasome [1,6,26]. Moreover, NQO1 was shown to control the stability of eukaryotic translation initiation factor 4GI against proteasomes and may, therefore, be involved in the regulation of mRNA translation [1,91].

### 3.5. Generation of NAD^+^ and β-Lapachone

β-lapachone, a redox-cycling quinone, has potent antitumor effects owing to its ability to produce ROS and oxidative DNA damage [26,92]. Furthermore, NQO1 has been shown to be the primary reductase responsible for the antitumor capacity of β-lapachone [93]. NQO1 catalyzes the conversion of β-lapachone to an unstable hydroquinone, which rapidly autoxidizes, producing high levels of ROS while simultaneously producing great amounts of NAD^+^ and NADP^+^ through the oxidation of NAD(P)H [26,93]. NQO1-mediated cycling of β-lapachone causes oxidative DNA damage, hyperactivation of poly (ADP-ribose) polymerase (PARP), disastrous depletion of NAD^+^ and ATP, and NAD^+^-keresis—a form of programmed necrosis [26,94,95,96,97]. These experiments revealed the opposing functions of NQO1 in β-lapachone-induced cytotoxicity, with roles in the production of reactive metabolites, while also generating NAD^+^ to facilitate DNA recovery [26]. Low doses of β-lapachone may be used to prevent oxidative DNA damage while increasing the NAD^+^:NADH ratio, thereby having potential applications in the management of various diseases. In studies using low doses of β-lapachone, the production of NAD^+^ to increase PARP and sirtuin-catalyzed reactions is thought to be involved in the therapeutic benefits of the treatment [26,98,99,100,101].

### 3.6. Colocalization with Microtubules

**Figure 3:**
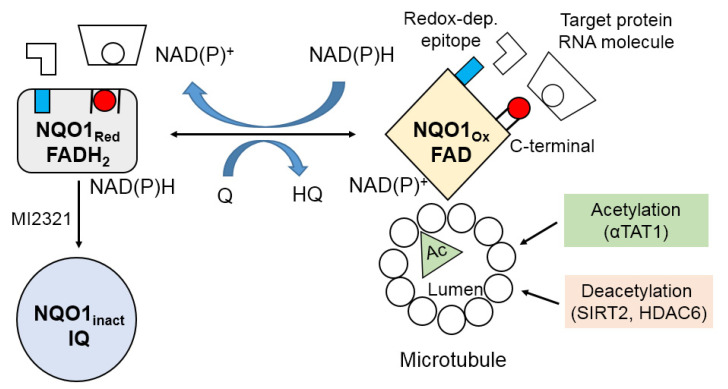
Schematic representation of NQO1 as a molecular redox switch via conformational changes. The conformational changes of NQO1 can be attributed to the levels of reduced pyridine nucleotides. Under physiological conditions, adequate levels of NAD(P)H maintain NQO1 in the reduced form (FADH_2_), preventing antibodies from binding to the C-terminal domain and redox-dependent epitope. When NAD(P)H levels decrease, however, NQO1 takes on an oxidized form (FAD), exposing its C-terminal tail and redox-dependent epitope. The conformational change in NQO1 in response to the intracellular NAD(P)^+^/NAD(P)H redox balance modifies the binding of either RNA molecules or target proteins to NQO1. NAD(P)H levels also induce NQO1 conformational changes during binding to microtubules. NQO1 inactivation by the indolequinone component of inhibitor MI2321 modifies the conformation of NQO1, blocking its binding to microtubules. Decreased levels of NQO1 bound to microtubules result in decreased deacetylation or increased acetylation of lysine^40^ in α-tubulin in the microtubular lumen. Abbreviations—Red: reduced; Ox: oxidized; inact.: inactivated; Q: quinone; HO: hydroquinone; IQ: indolequinone; Ac: acetylated; αTAT1: alpha-tubulin N-acetyltransferase; SIRT2: NAD-dependent deacetylase sirtuin 2; HDAC6: histone deacetylase 6.

Previous studies have shown that NQO1 colocalizes with α-tubulin to the centromere, midbody, and mitotic spindles [26,102,103]. Microtubules hold high levels of acetylated α-tubulin K40 on their luminal sides, and double-immunostaining studies have demonstrated that NQO1 colocalizes with these acetylated structures [102]. Binding of NQO1 dimers to microtubules may occur via their exposed positively charged C-terminal tails when NQO1 is in the oxidized state (Figure 3) [102,104]. Numerous NAD^+^-dependent enzymes, including PARP and sirtuin 2 (SIRT2), have also been shown to colocalize with these acetylated microtubule structures, indicating that NQO1 may provide NAD^+^ for these enzymes [26,105,106,107]. Recent reports have also demonstrated that the progression of mitosis is delayed when NQO1 is compromised [108]. Other studies showed that SIRT2 and NQO1 were bound and colocalized to the mitotic spindles, suggesting that NQO1 may act as a downstream regulator of SIRT2 deacetylase via its ability to provide NAD^+^ and bind to SIRT2 [26,108].

### 3.7. Control of mRNA Translation

In a study designed to capture proteins bound to mRNA, numerous FAD-containing redox enzymes, including NQO1, were identified as mRNA-binding proteins [6,109]. A recent study using ribonucleoprotein immunoprecipitation demonstrated that NQO1 binds to a subset of mRNAs in HepG2 cells, and that serine protease inhibitor (SERPIN) A1 mRNA—which encodes the SERPIN α-1-antitrypsin—is a target. This enzyme is related to various diseases, including chronic obstructive pulmonary disease, obesity-related metabolic inflammation, liver cirrhosis, and hepatocellular carcinoma [6,110]. NQO1 can bind the 3′-untranslated region and augment SERPINA1 translation. Although it is too early to establish the potential relevance of mRNA binding by NQO1, additional studies are needed in order to verify the specificity of binding, and to determine whether NQO1 plays broad roles in modulating translation [6]. 

## 4. Molecular Redox Switch and Conformational Changes in NQO1

In the structure of NQO1, the catalytically active form is produced from two engaging monomers, which form a homodimeric protein [6,8,18,111]. The active sites are located at the interface between the monomers, where they develop a noncovalent binding pocket for FAD [6,26]. NQO1 then undergoes a conformational change, discharging the oxidized pyridine nucleotide and developing an environment for quinone binding [17]. Owing to this Ping-Pong Bi–Bi kinetic mechanism, NQO1 may exist in the form of either a reduced (FADH_2_) or an oxidized (FAD) conformation, depending on the relative concentrations of the substrates and the reduced pyridine nucleotides (Figure 3) [6,104]. Subsequent studies have revealed that NQO1 exists in at least three conformational forms—i.e., oxidized, reduced, and inactivated forms—which have heterogeneous immunoreactivity to antibodies and, therefore, distinct implications for reacting with downstream proteins [26,104]. Biochemical proof of this conformational change in NQO1 was shown in studies demonstrating that the C-terminal domains of NQO1 were protected from proteolytic digestion following the binding of reduced pyridine nucleotides [6,112]. The conformational change in NQO1 in response to the binding of reduced pyridine nucleotides reveals that NQO1 could act as a molecular redox switch, whereby the three-dimensional shape of a protein can be regulated by the NAD(P)^+^/NAD(P)H redox balance. Similarly, the binding of NQO1 to *SERPINA1* mRNA has also been shown to be affected by the NAD(P)^+^/NAD(P)H redox balance [6,110]. This suggests that the pyridine nucleotide redox environment modulates the interaction of NQO1 with mRNA and proteins, and may act as a redox-dependent molecular switch to regulate the downstream cellular functions of NQO1 [6]. In summary, the pyridine nucleotide ratio seems to be essential for the downstream functions of NQO1, and is likely to regulate the interaction of NQO1 with other signaling proteins [6]. Moreover, these findings highlight the redox- and conformation-dependent transformations in the NQO1 structure, and provide an explanation for the alternative redox-switching and/or redox-sensing roles of NQO1 [26]. Alterations in pyridine nucleotide redox status can lead to changed NQO1 conformation, leading to regulation of downstream NQO1 interactions. However, it is still unclear whether conformational changes in NQO1 lead to alterations in the localization of the protein. Although NQO1 is a predominantly cytosolic enzyme, it has also been shown to have substantial mitochondrial, nuclear, and membrane pools in different cell types [26,113,114,115,116].

## 5. Genetic Polymorphisms in *NQO1* and Disease

The potential roles of NQO1 in health and illness have also been widely studied in human populations [1,6,26]. In humans, most studies have evaluated its activity in tissues from patients with various diseases, as well as changes in the NQO1 enzyme at the protein and mRNA levels, whereas epidemiological or clinical studies have evaluated the associations between disease risk and NQO1 gene polymorphisms [1]. In this context, two genetic variants have been verified in NQO1, i.e., NQO1*2 (C609T; proline to serine) and NQO1*3 (C465T; arginine to tryptophan). The *NQO1* wild-type allele is designated as NQO1*1 [1,26].

Over 20 single-nucleotide polymorphisms (SNPs) have been detected in *NQO1* [6,117]. The most common SNP of NQO1 is a C-to-T substitution at nucleotide position 609 of the NQO1 cDNA (rs1800566), also known as the NQO1*2 mutant allele [118]. This nucleotide variation is responsible for the exchange of proline to serine amino acid at position 187 (P187S), which is accompanied by a decrease in enzyme action due to the variability of the protein product [6,118]. The prevalence of this variant differs broadly by ethnicity, and a relationship has been described between the presence of variant alleles [6,119]. The interethnic variances in this gene have been reported to range from approximately 15% to 50% in Caucasian to Chinese populations, respectively, and the frequency of NQO1*2/*2 homozygosity is documented to range between approximately 2% and 20% in diverse racial groups [6,119,120]. The mutant NQO1*2 protein is extremely unstable because of a decreased affinity for the FAD cofactor, and is easily ubiquitinated by the proteasome [6,121,122]. Therefore, the activity of the homozygous variant genotype (NQO1*2/*2) enzyme is substantially undetectable, whereas NQO1*1/*2 heterozygotes have nearly intermediate activities between the homozygous polymorphism genotype and the wild type (NQO1*1/*1) [1,118,119,123,124].

The second most common SNP of NQO1 is a replacement of a single nucleotide from C to T at nucleotide position 465 (rs4986998), also referred to as NQO1*3, which is responsible for the exchange of the amino acid at codon 139 from arginine to tryptophan (R139W) [6,125,126]. The prevalence of NQO1*3 SNP is generally low, and ranges from 0% to 5% among diverse ethnic populations [1,6,119]. This polymorphism is similar to the wild-type NQO1*1, resulting in substitutional mRNA splice sites that can result in a deletion of exon 4 and produce a protein deficient in quinone-binding sites, for which enzyme activity differs depending on the substrate [6,119]. Recently, seven NQO1-mutant transcripts were recognized, of which six encoded potential NQO1 isoforms [6,127]. The observed increases in the expression of exon-deleted NQO1 mutants provided a reasonable description for the reduced enzyme activity of NQO1*3 polymorphism [128].

## 6. NQO1 and Disease

OS mediated by increased ROS production leads to an imbalance in redox pathways, followed by oxidative damage [19]. This is a major factor in the pathophysiology of various acute and chronic diseases. In particular, pathophysiological manifestations of cardiovascular diseases—such as heart failure, atherosclerosis, cardiac hypertrophy, hypertension, and diabetes—may be induced by OS [19,20,129,130]. Here, we focus on the potential roles of NQO1 in atherosclerosis and cardiovascular diseases, insulin resistance and diabetes mellitus, metabolic syndrome, aging, and AD, as summarized in Table 1.

### 6.1. Atherosclerosis and Cardiovascular Diseases

Under basal conditions, NQO1 is abundantly expressed in the cardiovascular system [1,65,79,80]. The expression of NQO1 in cardiac and vascular cells and tissues can also be induced by various stimuli, including cigarette smoke, oxidants, sheer force, inflammatory cytokines, reactive aldehydes, chemoprotective agents, homocysteine, and statins [1,130]. Thus, overexpression of NQO1 may be an adaptive protection mechanism against inflammation and OS. Indeed, overexpression of NQO1 in cultured vascular smooth muscle and endothelial cells protected the cells against pro-inflammatory cascades and oxidative cytotoxicity activated by cytokines [1,131,145]. A previous work revealed that β-lapachone-induced activation of NQO1 inhibits carotid artery restenosis via suppression of the proliferation of vascular smooth muscle cells in a balloon injury model of the rat carotid artery [132]. Experimental upregulation of NQO1 by adenovirus vectors in cultured vascular cells also reduces the expression of hyperglycemia-mediated endothelial adhesion molecule and tumor necrosis factor-α, and blocks smooth muscle cell migration [131,145,146]. Taken together, these in vitro and in vivo findings suggest that NQO1 may act as an important defender against atherogenesis, and that gene delivery or pharmacological induction of NQO1 in the vascular or circulatory systems may, therefore, represent a valuable strategy for managing vascular diseases [1]. 

A previous epidemiological study reported that patients with the NQO1 C609T polymorphism (NQO1*2 genotype) showed a greater prevalence of atherosclerotic carotid plaques than those without this genotype in type 2 diabetic patients [1,133]. Additionally, studies in patients undergoing coronary bypass graft surgery demonstrated a higher inflammatory response, as supported by elevated interleukin-6 levels, in patients with the NQO1*2 polymorphism compared with patients without this polymorphism [1,147]. Moreover, significantly greater levels of C-reactive protein—a representative biomarker for atherosclerosis and coronary health—were also reported in patients with coronary artery disease combined with a lack of activity of NQO1 [134], suggesting a protective role of NQO1 against inflammation in the cardiovascular system. A previous study in patients with ischemic stroke associated with large-artery atherosclerosis showed that NQO1*2 polymorphisms were connected with a lower risk of atherosclerosis-associated stroke [148]. Furthermore, the reduced thrombogenic risk was thought to be related to the decreased metabolic activity of vitamin-K-dependent coagulation factors. In this regard, NQO1 has been known to catalyze the reduction of vitamin K to its active form—vitamin K hydroquinone. Thus, the role of NQO1 in the cardiovascular system may vary depending on the specific disease conditions [1,148]. 

### 6.2. Insulin Resistance and Diabetes Mellitus

The Keap1/Nrf2 pathway is a promising target for the management of metabolic syndrome and type 2 diabetes mellitus [26,149]. Genetic activation of Keap1/Nrf2 in diabetic animal models has been shown to alleviate insulin resistance and to prevent the onset of diabetes mellitus [150]. Multiple biological pathways and mechanisms are associated with actions of Nrf2, including protection against OS, metabolism of glycogen, repression of hepatic gluconeogenesis, functions of mitochondria, synthesis of lipids, inflammation, and crosstalk with other molecular pathways [26,149,150,151,152,153,154]. Pharmacological activation of Nrf2 by an acetylenic tricyclic bis(cyano enone), TBE-31, was also shown to reverse insulin resistance in obese mice, and to inhibit hepatic steatosis and fibrosis via suppression of endoplasmic reticulum, oxidative, and inflammatory stresses [155]. However, these Keap1/Nrf2 pathways are complex processes, and emerging experimental data have recently been published regarding the conflicting roles of Nrf2 in adipogenesis, insulin resistance, obesity, insulin sensitivity, and inflammation—partly because of the diversity of Nrf2-mediated functions [26,156,157].

More recently, an interesting study has shown that the astaxanthin (Ast; 3,3′-dihydroxy-β,β′-carotene-4,4′-dione)—a type of red carotenoid—reduces OS in diabetic animal models induced by streptozotocin combined with high fat and high sugar intake. Of 13,784 diabetes mellitus targets and 120 Ast-targeted proteins evaluated in a combined network/transcriptional analysis, NQO1 was identified as one of three major targets, together with collagen type V alpha-1 and neurogenic locus notch homolog protein 2. These three Ast-intersecting targets finally showed a systematic overview of how Ast works in multiple pathways to reduce Ast-induced OS and insulin resistance [26,135]. The association between the NQO1*2 genotype and risk of diabetes mellitus has also been studied in a Danish population and a Chinese population, and no correlation between diabetes and NQO1*2 polymorphism was detected in either study [1,136,137].

### 6.3. Metabolic Syndrome

NQO1, a downstream target of Nrf2, seems to play a protective role in metabolic syndrome. The onset of metabolic syndrome is accompanied by pro-inflammatory and oxidative processes, which cause insulin insensitivity and disease progression [26,158,159]. NQO1-null mice exhibit higher NAD(P)H/NAD(P)^+^, higher levels of hepatic triglycerides, and significantly lower levels of abdominal adipose tissue compared to wild-type mice. Insulin tolerance tests revealed that NQO1-null mice were insulin resistant [1,138]. Thus, NQO1 may exert protective effects on central adiposity, dyslipidemia, and insulin resistance—representative key features of metabolic syndrome. In murine models of metabolic syndrome, β-lapachone-induced pharmacological activation of NADH oxidation by NQO1 showed the substantial improvement of metabolic syndrome and related phenotypes, including obesity, fatty liver, dyslipidemia, and glucose intolerance [1,101]. β-Lapachone is a high-affinity natural substrate specific to NQO1, and can vigorously induce the oxidation of NADH by NQO1 in vivo and in vitro [1,56,160]. These observational findings suggest that genetic as well as pharmacological therapeutic strategies targeting NQO1 may have applications in the management of metabolic syndrome [1,101]. 

Various human studies have demonstrated that the *NQO1* gene is extremely expressed in adipocytes, and its transcriptome can be decreased via diet-associated weight reduction [1,139]. The expression of NQO1 mRNA has also been shown to be positively correlated with glucose tolerance, adiposity, and markers of liver dysfunction, indicating a possible association of NQO1 with the metabolic complications of obesity. However, it is still unclear whether NQO1 play etiological roles in the pathophysiology of obesity and metabolic complications, or whether increased NQO1 mRNA expression may be caused by obesity. Alternatively, upregulation of NQO1 could be an adaptive physiological response to human obesity and related complications, and may act as a protective therapeutic tool [1,139]. 

### 6.4. Aging

The level of NQO1 decreases with age, and decreased NQO1 provides less efficient protection from OS [26,140,161]. One of the more vigorous experimental methods for modulating aging rates in animal models is calorie restriction (CR), which causes upregulation of NQO1 and the plasma membrane redox system (PMRS) in the brain and liver, thereby conferring protection against OS [26,117,161,162]. Overexpression of two NAD^+^-generating enzymes—NQO1, and cytochrome b5 reductase—mimics CR in animal models [163]. CR, however, improves the survival of NQO1-null mice, demonstrating that although NQO1 may contribute to the effect of CR, it is also not required in order to achieve the beneficial effect of CR on longevity [26,164]. Feeding mice with β-lapachone to regulate NAD^+^/NADH inhibits age-dependent decreases in cognitive and motor function in aged mice [26,100]. More recently, a previous study showed that augmentation of cellular NAD+ levels by β-lapachone effectively inhibited aging-related hearing loss and accompanying deleterious effects in a murine model [101,140]. 

### 6.5. Alzheimer’s Disease 

NQO1 is correlated with early pathological alterations in AD, and expression of NQO1 is associated with the localization and progression of human brain pathology in patients with AD [26,141,142,165]. NQO1 expression is increased in the brains of patients with AD when compared with age-matched controls, and increased expression of NQO1 results in the accumulation of NQO1 in brain regions affected by AD pathology [26,141]. Elevated NQO1 has also been detected in the hippocampus, and in neurofibrillary tangles in postmortem samples from patients with AD, but not from the brains of younger patients, or in age-matched controls without AD [165]. Additionally, NQO1 immunostaining revealed the localization of NQO1 adjacent to senile plaques [141]. The NQO1*2 (NQO1 C609T) polymorphism is even more prevalent in Asian populations [26,119,123,127], and a previous meta-analysis demonstrated that this polymorphism significantly increases the risk of AD in Chinese populations [143]. Furthermore, a recent review demonstrated that understanding the association between AD pathology and NQO1 may improve our insights into the pathogenesis of the disease, and could lead to the development of novel therapeutic approaches for the management of AD [166]. Finally, studies of NQO1 and NQO1*2 as model flavoenzymes showed that these enzymes could bind together with β-amyloid (1-42) fibrils, which are known to be key elements of amyloid plaques in AD patients [26,144]. 

## 7. Perspectives

The development of NQO1-targeted drug delivery and novel compounds activated by NQO1 has been a focus area for active research [26]. In recent studies, the use of NQO1 to regulate the NAD^+^/NADH redox balance has been explored chiefly through β-lapachone. NQO1 effectively reduces β-lapachone to a hydroquinone form, which is quickly autoxidized back to a quinone form while generating oxidized pyridine nucleotides (NAD^+^ and NADP^+^) and consuming reduced pyridine nucleotides (NADH and NADPH) [6,56]. Therefore, the strategy to freely regulate the NAD(P)^+^/NAD(P)H redox balance ratio via NQO1-mediated metabolism may have therapeutic potential in the future [6]. While it is yet unclear as to how to target NAD metabolism as a management approach for aging, the use of NAD^+^ precursors to upregulate cellular NAD^+^ levels has led to improvements in aging-related and cardiovascular functional deficits [26,167,168,169,170]. Intriguingly, a recent high-throughput anti-aging drug test revealed two compounds in more than 2600 screening tests, both of which influenced pyridine nucleotides’ redox ratio, and one of which functioned through the catalytic action of NQO1 [171]. Further studies, however, are needed in order to fully elucidate the roles of NQO1 in the aging phenotypes, considering the multiple suggested roles of NQO1 to date [26]. Currently, air pollution is a global risk factor for public health, and it will pose a more serious problem in the future. Numerous studies have revealed that the exposure to fine particulate matter (PM)—especially at a diameter smaller than 2.5 μm—is correlated with diverse health-related outcomes, such as chronic obstructive pulmonary disease, asthma, cardiovascular diseases, AD, stroke, and even premature death [130,172]. PM is associated with airway oxidative stress and inflammation, causing the activation of Nrf2 and elevation of NQO1. Increased levels of NQO1 in the airways may be harmful or beneficial, because it plays a dual role as an activator of pre-carcinogens or a phase-II detoxification enzyme, respectively. Additional study is required in order to more meticulously characterize the role of the NQO1- and Nrf2-mediated OS response in PM-mediated toxicity in the future [130,172]. In terms of recent cancer therapy, current therapeutic goals are to treat cancer with high efficacy but with minimal harmful systemic effects, by targeting overexpressed proteins found in tumor cells. A representative example of such an approach is photodynamic therapy (PDT)—a noninvasive antitumor strategy, targeting or activating NQO1 [173,174]. There may be three options for NQO1-mediated PDT: The first is the discovery or synthesis of more efficacious derivatives or analogs of β-lapachone than its conventional form. The second option is the development of PDT probes activating NQO1 effectively as a potent photosensitizer (PS). The last option is the combined approach of the first and second options [173,174]. With the improvement of nanotechnology, the clinical application of PDT in deep-located tumors has become promising. Novel nanoparticles targeting NQO1 are able to act as better PS carriers, can rearrange the PS to a higher absorption peak, and work better in a hypoxic tumor microenvironment. Moreover, NQO1-targeted PDT combined with other strategies—such as immunotherapy, gas therapy, and starvation therapy—can stimulate immune reaction, augment the therapeutic effect of ROS, and deteriorate cancer cells, respectively [174,175].

## 8. Conclusions

NQO1 plays a crucial role in quinone metabolism, facilitating the production of hydroquinones with different chemical properties to those of their maternal quinones. The stability of the converted hydroquinone is an important factor in determining whether NQO1 detoxifies a reactive quinone or augments cellular toxicity. NQO1 was originally classified as a detoxifying enzyme, but it is now known to have many biological functions other than quinone metabolism [6]. Supplying a cellular source of NAD^+^ that may be critical for inhibiting or preventing metabolic syndrome, binding to mRNA to enhance protein translation, and protecting proteins from proteasomal degradation represent some of the newly discovered functions of NQO1. The effect of null polymorphism of NQO1 on CoQ metabolism has not yet been fully elucidated, but the role of NQO1 as a crucial element of the PMRS, and its ability to reduce CoQ, indicate the essential antioxidant properties of this enzyme. Intriguingly, the capacity of NQO1 to bind to RNA and proteins depends on the redox ratio of pyridine nucleotides, indicating that this protein can function as a redox-dependent switch [6]. Studies of gene polymorphisms and experimental models have suggested that this protein may play important roles in cardiovascular injury and related conditions, such as metabolic syndrome and atherogenesis [1]. Additional studies are required in order to determine the underlying mechanisms and protective effects of NQO1 in various types of cardiovascular disease. In this context, continued mechanistic studies of the molecular regulation and biochemical activities of NQO1 will present additional insights into the importance of this phase-II protein in health and cardiovascular disease [1].

## Figures and Tables

**Table 1:** Summary of studies on NQO1 and various disease entities.

Author	Published (Year)	Role of NQO1	Reference
**Atherosclerosis and Cardiovascular Diseases**			
Lee, et al.	2007	Reduced TNF-α-induced migration of human VSMCs	[131]
Kim, et al.	2009	Prevented arterial restenosis	[132]
Han, et al.	2009	C609T variant was associated with carotid artery plaques in T2DM patients	[133]
Isbir, et al.	2008	C609T variant was related to higher IL-6 levels	[133]
Martin, et al.	2009	Lack of activity of NQO1 was associated with elevation of CHD and CRP	[134]
**Insulin Resistance and Diabetes Mellitus**			
Sun, et al.	2020	Astaxanthin reduced oxidative stress and insulin resistance	[135]
Wang, et al.	2006	C609T variant was not associated with DM in Chinese subjects	[136]
Kristiansen, et al.	1999	C609T variant was not related to DM in Danish subjects	[137]
Gaikward, et al.	2001	NQO1-null mice were insulin resistant	[138]
**Metabolic Syndrome**			
Hwang, et al.	2009	β-Lapachone showed improvement of metabolic syndrome	[101]
Gaikward, et al.	2001	NQO1-null mice exhibited higher NAD(P)H/NAD(P)^+^, higher TG level, and lower abdominal fat	[138]
Palming, et al.	2007	NQO1 was correlated with adiposity and liver dysfunction	[139]
**Aging**			
Lee, et al.	2012	β-Lapachone prevented health decline in aged mice	[100]
Kim, et al.	2019	β-Lapachone improved age-related hearing impairment	[140]
**Alzheimer’s Disease**			
SantaCruz, et al.	2004	NQO1 was located adjacent to senile plaques	[141]
Wang, et al.	2000	NQO1 activity was increased in AD	[142]
Luo, et al.	2016	C609T variant was associated with risk of AD in Chinese subjects	[143]
Martinez-Limon, et al.	2016	NQO1 could bind together with β-amyloid fibrils	[144]

Abbreviations—TNF: tumor necrosis factor; VSMCs: vascular smooth muscle cells; T2DM: type 2 diabetes mellitus; IL: interleukin; CHD: coronary heart disease; CRP: C-reactive protein; DM: diabetes mellitus; TG: triglyceride; AD: Alzheimer’s disease.

## Data Availability

Data sharing not applicable.
